# CXCR2 perturbation promotes Staphylococcus aureus implant-associated infection

**DOI:** 10.1099/jmm.0.001821

**Published:** 2024-04-03

**Authors:** Mike Akaraphanth, Tara M. Nordgren, Casey M. Gries

**Affiliations:** 1 School of Medicine, University of Colorado, Aurora CO 80045, USA; 2 Department of Environmental and Radiological Health Sciences, Colorado State University, Fort Collins CO 80523, USA; 3 Department of Microbiology, Immunology and Pathology, Colorado State University, Fort Collins CO 80523, USA

**Keywords:** biofilm, CXCR2, neutrophil, *Staphylococcus aureus*

## Abstract

**Introduction.***Staphylococcus aureus* is the leading cause of acute medical implant infections, representing a significant modern medical concern. The success of *S. aureus* as a pathogen in these cases resides in its arsenal of virulence factors, resistance to multiple antimicrobials, mechanisms of immune modulation, and ability to rapidly form biofilms associated with implant surfaces. *S. aureus* device-associated, biofilm-mediated infections are often persistent and notoriously difficult to treat, skewing innate immune responses to promote chronic reoccurring infections. While relatively little is known of the role neutrophils play in response to acute *S. aureus* biofilm infections, these effector cells must be efficiently recruited to sites of infection via directed chemotaxis. Here we investigate the effects of modulating CXC chemokine receptor 2 (CXCR2) activity, predominantly expressed on neutrophils, during *S. aureus* implant-associated infection.

**Hypothesis.** We hypothesize that modulation of CXCR2 expression and/or signalling activities during *S. aureus* infection, and thus neutrophil recruitment, extravasation and antimicrobial activity, will affect infection control and bacterial burdens in a mouse model of implant-associated infection.

**Aim.** This investigation aims to elucidate the impact of altered CXCR2 activity during *S. aureus* biofilm-mediated infection that may help develop a framework for an effective novel strategy to prevent morbidity and mortality associated with implant infections.

**Methodology.** To examine the role of CXCR2 during *S. aureus* implant infection, we employed a mouse model of indwelling subcutaneous catheter infection using a community-associated methicillin-resistant *S. aureus* (MRSA) strain. To assess the role of CXCR2 induction or inhibition during infection, treatment groups received daily intraperitoneal doses of either Lipocalin-2 (Lcn2) or AZD5069, respectively. At the end of the study, catheters and surrounding soft tissues were analysed for bacterial burdens and dissemination, and *Cxcr2* transcription within the implant-associated tissues was quantified.

**Results.** Mice treated with Lcn2 developed higher bacterial burdens within the soft tissue surrounding the implant site, which was associated with increased *Cxcr2* expression. AZD5069 treatment also resulted in increased implant- and tissues-associated bacterial titres, as well as enhanced *Cxcr2* expression.

**Conclusion.** Our results demonstrate that CXCR2 plays an essential role in regulating the severity of *S. aureus* implant-associated infections. Interestingly, however, perturbation of CXCR2 expression or signalling both resulted in enhanced *Cxcr2* transcription and elevated implant-associated bacterial burdens. Thus, CXCR2 appears finely tuned to efficiently recruit effector cells and mediate control of *S. aureus* biofilm-mediated infection.

## Background

*Staphylococcus aureus* is a leading cause of both healthcare- and community-associated infections, often manifesting as skin and soft tissue infections, sepsis, endocarditis and osteomyelitis [1]. Infection risk is increased in those with an implanted medical device, such as an orthopaedic prosthesis, cardiac device or indwelling catheter. These surfaces are rapidly coated with plasma and extracellular matrix proteins that modulate a foreign body reaction but also present a target for bacterial attachment and biofilm formation [2]. As opposed to a planktonic lifestyle, biofilms are adherent communities of bacteria encased within a self-produced matrix of extracellular DNA, proteins and/or polysaccharides. Host-induced three-dimensional biofilm architecture not only poses a physical barrier to immune cell infiltration and phagocytosis, but biofilm products actively skew host inflammatory responses, enabling infection persistence [3,4]. Thus, with the rise of antibiotic resistance limiting therapeutic options, further understanding of immune responses associated with *S. aureus* biofilm infections is essential.

Neutrophils are the most abundant leucocyte in the blood and the first line of cellular immune defence against invading pathogens [5]. Neutrophils migrate along chemokine gradients to sites of inflammation where they can prevent infection through phagocytosis, degranulation, production of reactive oxygen species, and neutrophil extracellular traps [5]. Many of these inflammatory responses are regulated by CXC chemokines, a family of small protein ligands with strong activation and chemotactic activity toward neutrophils. CXC chemokine receptors, such as CXCR2, interact with these ligands and activate neutrophils through G-protein coupled receptors and their corresponding second messenger systems [6]. The CXCR2 signalling axis has proven to be crucial in several models of bacterial infection [7,11]; however, previous observations have shown that a remarkably small number of neutrophils are recruited to sites of *S. aureus* biofilm-mediated infection [12]. It is posited that the lack of infiltrating neutrophils, likely through chemotactic inhibition or direct killing, contributes significantly to *S. aureus* biofilm establishment and persistence [12,13]. Using intravital multiphoton microscopy of a mouse implant model of *S. aureus* infection, we have previously demonstrated a significant increase in neutrophil migration velocity and displacement proximal to the infection, indicative of cellular activation [14]. However, increased neutrophil meandering, a measure of migration directionality, demonstrated that many neutrophils are redirected away from the biofilm.

Remarkably little is known of the role CXCR2 plays during *S. aureus* infection, and the role of CXCR2 during biofilm-mediated infection has not been investigated. Several pieces of prior evidence point to the importance of CXCR2 in *S. aureus* planktonic infections. In one report, CXCR2 inhibitors resulted in decreased neutrophil accumulation and pro‐inflammatory mediator production in a mouse model of *S. aureus* septic arthritis [9]. CXCR2 has also been identified as a cellular target for *S. aureus* ɣ-hemolysin AB [15], leukotoxin ED [15] and Staphopain A^15^, highlighting the important role CXCR2 likely plays during infection. Finally, *Cxcr2* knockout mice exhibited impaired neutrophil extravasation and increased bacterial burdens in a *S. aureus* brain abscess model [16]. While a lack of CXCR2 appears to worsen infection, it is unknown if augmenting CXCR2, and therefore potentially improving neutrophil migration, might prove an effective means of promoting infection clearance. Here, we examine the effects of modulating CXCR2 activity during *S. aureus* implant-associated infection by utilizing two distinct therapeutic mechanisms of perturbing CXCR2 signalling; lipocalin-2 (Lcn2), a secreted innate immune protein previously shown to induce neutrophil CXCR2 expression [17], and AZD5069, a potent and selective CXCR2 antagonist proven useful in inhibiting neutrophil chemotaxis in mice [18]. The overarching goal of this study is to determine if *S. aureus* immunomodulation could be combated with therapeutic modification of CXCR2 activity, as this may lead to the identification of a novel therapeutic strategy to combat biofilm-mediated infections.

## Methods

### *S. aureus* strain and culture conditions

The *S. aureus* strain used in these studies is a USA300 community-associated MRSA skin and soft tissue infection isolate cured of plasmid p03 [19], referred to as LAC in this work. Isolated colonies on tryptic soy agar (TSA) with 5 % sheep blood (Remel, USA) were cultured in 25 ml brain heart infusion broth (BD, USA) for 16 h at 37 °C, 250 r.p.m. and washed twice with PBS (Cytiva, USA). Culture density (OD_600_) was measured to estimate appropriate bacterial dilution prior to infection.

### Mouse flank catheter model of *S. aureus* infection

The animal work in this study was carried out in strict accordance with the recommendations in the Guide for the Care and Use of Laboratory Animals of the National Institutes of Health, the Animal Welfare Act, and USA federal law. The protocol was approved by the Colorado State University Institutional Animal Care and Use Committee (IACUC). Indwelling catheter-associated biofilm infections were performed as previously described [3]. Briefly, 8 week wild-type C57BL/6 mice (Jackson Labs, USA) were anesthetized with ketamine-xylazine (100 mg/kg-5 mg/kg of body weight), and the surgical site was shaved and disinfected with povidone-iodine plus 70 % ethanol. A 1 cm subcutaneous incision was made in the mouse’s left flank, and a blunt instrument was used to create a pocket for inserting a 1 cm sterile 16-gauge Teflon-coated intravenous catheter (Excel International, USA). The incision was then sutured and sealed using Vetbond tissue adhesive (3M, USA). Then, 10^3^ CFU of *S. aureus* LAC in 20 µl PBS was injected directly into the catheter lumen using a 29-gauge insulin syringe (BD, USA). The study duration lasted for 5 days, with treatment and vehicle dosing provided daily starting immediately after implant/infection and ending the day prior to sacrifice.

Treatment groups received daily intraperitoneal (IP) injections of either lipocalin-2 (Lcn2; Sino Biological, USA) diluted to working concentrations for 50 ng g^−1^, 100 ng g^−1^ or 150 ng g^−1^ doses in physiological water (PW; InvivoGen, USA) or selective CXCR2 antagonist AZD5069 (MedChemExpress, USA) diluted to working concentrations for 50 µg g^−1^ and 100 µg g^−1^ doses in 45 % PW, 40 % PEG-300, 10 % DMSO, 5 % Tween-80. Control groups received daily IP injections of respective vehicle solutions. The Lcn2 doses were chosen based on studies showing up to twofold increased serum Lcn2 levels at 150 ng g^−1^ [20], while the lowest dose was chosen based on studies showing its influence on encephalomyelitis disease severity [21]. The doses of AZD5069 were chosen based on studies showing that 100 µg g^−1^ could antagonize CXCR2 and effectively reduce murine tumour size [18].

At the end of the study duration, mice were sacrificed with CO_2_ inhalation and cervical dislocation. For post-infection tissue collection and bacterial enumeration, catheters were removed and placed in 0.1 ml PBS on ice prior to vortexing and sonication to dissociate bacteria from the catheter surface. Spleens were collected and flash frozen. Soft tissue surrounding the catheter was collected, and a small (~10 mg) portion placed in 100 µl of RNAprotect Tissue Reagent (Qiagen, Germany). The remaining tissue was weighed using an analytical balance and dissociated using Pellet Pestles (Fisher, USA) in 0.5 ml PBS containing 1 x EDTA-free protease inhibitor cocktail (Roche, Switzerland) on ice. Bacterial titres were quantified on TSA with 5 % sheep blood and expressed as CFU per millilitre for catheters or CFU per gram of tissue. IL-10 was quantified in soft tissue homogenates using the mouse DuoSet ELISA (R&D Systems, USA) according to manufacturer recommendations.

In some cases, small portions of infected tissues were collected for histology and stored in 10 % normal buffered formalin. Fixed tissues were processed, and sections were H&E stained at the CSU Experimental Pathology facility. Slides were scanned at 20× magnification on the VectraPolaris system (Akoya Biosciences, Marlborough, MA) and Phenochart software was used for image acquisition and analysis.

### Quantitative real-time PCR

Total tissue RNA was collected from RNAprotect stabilized tissues using an RNeasy kit (Qiagen, Germany). Tissues were disrupted in Lysing Matrix S (MP Biomedicals, USA) tubes containing 0.6 ml Buffer RLT using a Bead Ruptor (Omni International, USA). cDNA was generated using Superscript IV reverse transcriptase (Invitrogen, USA) and quantitative real-time PCR (qPCR) was performed using TaqMan Fast Advance Master Mix with primer-probes specific for *Gapdh* (Mm99999915_g1) and *Cxcr2* (Mm00438258_m1) (Applied Biosystems, USA).

### Statistical analyses

Significant differences between experimental groups were determined as described in the respective figure legends. GraphPad Prism 10 (GraphPad, USA) was used for all graphing and statistical analysis calculations, and a *P* value of <0.05 was considered statistically significant.

## Results

### Lipocalin-2 promotes the spread of *S. aureus* from biofilm into soft tissue

**Fig. 1.**
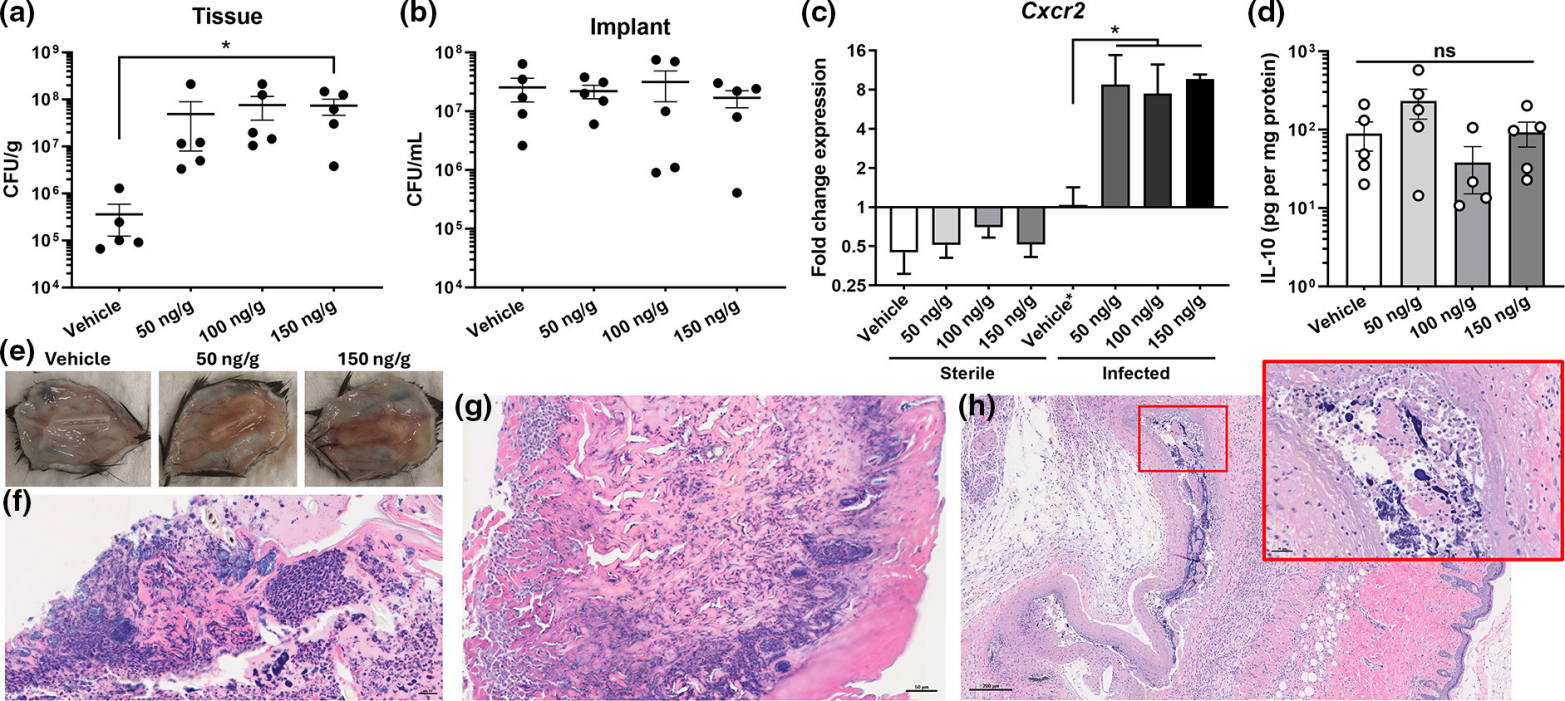
Lcn2 promotes *S. aureus* biofilm infection. Implanted catheters were either kept sterile or infected with *S. aureus*, and mice were treated with 0 (vehicle), 50, 100, or 150 ng g^−1^ Lcn2 daily for 5 days. Bacterial burdens associated with the catheter and surrounding soft tissue were quantified (**a, b**). RNA was isolated from the soft tissues and *Cxcr2* expression was quantified (**c**). IL-10 in soft tissue homogenate supernates was quantified via ELISA (**d**). Representative images for gross tissue pathology (e) and H&E staining for histopathology in vehicle (**f**), 50 ng g^−1^ (**g**), and 150 ng g^−1^ (**h**) treated animal tissues are shown. Data are from three independent experiments. Horizonal lines in panels (**a)** and (**b)** indicate mean colony forming units (CFUs) with standard deviation shown. Each symbol denotes an individual mouse. Statistical analyses were performed using one-way ANOVA with Dunnett post-hoc analysis. *P<0.05.

To elucidate the impact of augmented CXCR2 induction during *S. aureus* infection of a subcutaneous indwelling catheter, mice were administered daily intraperitoneal doses of 50, 100, or 150 ng g^−1^ Lcn2. After 5 days, bacterial titres associated with the catheter and surrounding soft tissue were determined. Mice receiving any dose of Lcn2 developed higher bacterial burdens in catheter-associated tissues compared to vehicle-treated animals, while no observable changes were found in bacteria burdens associated with the implants themselves (Fig. 1a, b). To assess the efficacy of our Lcn2 treatment strategy, *Cxcr2* transcription was quantified using RNA isolated from the soft tissue of mice harbouring either infected or sterile catheter implants. We observed a twofold increase in *Cxcr2* transcription in implant-associated tissues from infected compared to sterile mice (Fig. 1c), indicating an increased presence of CXCR2-expressing cells at active infection sites. Furthermore, *Cxcr2* expression in tissues associated with sterile implants was unchanged in mice receiving any dose of Lcn2, revealing that recruitment of CXCR2-expressing cells was dependent on infection. In the infected implant mice, all Lcn2 doses resulted in a significant, approximately eightfold *Cxcr2* induction within the tissues compared to vehicle-control animals. This upregulation appeared isolated to infected tissues, as no differences were observed in spleens collected from all animals (data not shown).

To further assess the immunological response to infection with and without Lcn2 treatment, we first quantified tissuecombat their antimicro levels of IL-10, an important determinant of *S. aureus* implant infection outcomes [4,22]. As shown in Fig. 1d, no significant differences were found among vehicle and Lcn2 treatment groups. Consistent with infection titres, Lcn2 treatment resulted in marked increases in soft tissue accumulation surrounding the infected implanted catheter (Fig. 1e). Histopathological analyses of these tissues revealed evidence of streaming neutrophilic debris, NETosis, tissue damage and fibrosis proximal to visible biofilm and/or implant site (Fig. 1f–h). Together, these results demonstrate that *S. aureus* infection recruits CXCR2-expressing cells, and that Lcn2-mediated enhancement of *Cxcr2* induction during implant infection promotes bacterial invasion from the biofilm into surrounding soft tissues.

### CXCR2 antagonist AZD5069 promotes *S. aureus* biofilm infection development

**Fig. 2.**
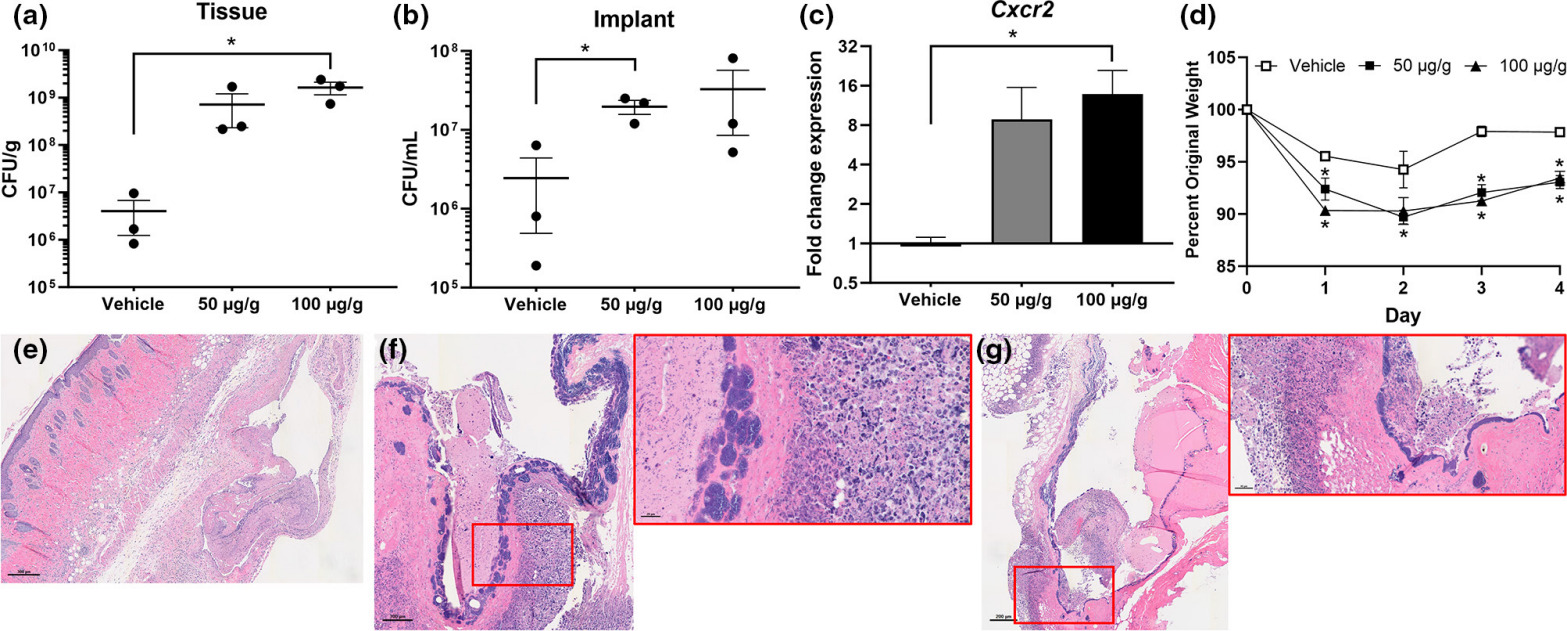
CXCR2 antagonist worsens *S. aureus* implant infection. Implanted catheters were infected with *S. aureus*, and mice were treated with 0 (vehicle), 50 or 100 µg g^−1^ AZD5069 daily for 5 days. Bacterial burdens associated with the catheter and surrounding soft tissue were quantified (**a, b**). RNA was isolated from the soft tissues and *Cxcr2* expression was quantified (**c**). Animal weights during the study were recorded as a measure of health (**d**). Representative images of H&E staining for histopathology in vehicle (**e**), 50 µg g^−1^ (**f**), and 100 µg g^−1^ (**g**) treated animal tissues are shown. Data are from two independent experiments. Horizonal lines in panels (**a)** and (**b)** indicate mean CFUs with standard deviation shown. Each symbol denotes an individual mouse. Statistical analyses were performed using one-way ANOVA with Dunnett post-hoc analysis. *P<0.05.

Given our findings of higher bacterial burdens in the context of enhanced CXCR2 levels, we next sought to determine if inhibition of CXCR2 signalling would alter implant-associated *S. aureus* infection outcomes by administering daily doses of 50 or 100 µg g^−1^ AZD5069. Compared to vehicle control mice, AZD5069 treatment markedly increased bacterial burdens associated with both the indwelling catheter and surrounding soft tissues (Fig. 2a, b). We assessed *Cxcr2* expression in tissues collected from vehicle- and AZD5069-treated mice and, as shown in Fig. 2c, AZD5069 treatment resulted in 8- to 14-fold increases in *Cxcr2* expression. Infected mice receiving AZD5059 also showed significantly increased weight loss over the course of the study, compared to vehicle treatment (Fig. 2d). Finally, histopathological analyses of infected tissues from AZD5069-treated animals revealed evidence of robust fibrosis and chronic inflammation proximal to visible biofilm and implant site (Fig. 2e–g). Furthermore, compared to vehicle, extensive evidence of biofilm was visible along the catheter implant region in AZD5069-treated animals. These biofilms were separated from immune cell infiltration by thick (≥30 µm) walls of fibrosis and inflammation, likely responsible for the increase in both tissue- and implant-associated *S. aureus* titres. These data demonstrate that inhibition of CXCR2 activity results in severe *S. aureus* infection outcomes, including both increased implant-associated titres and invasion into surrounding soft tissues.

## Discussion

Neutrophils play an essential role in the innate immune response to bacterial infection, and *S. aureus* has evolved a number of ways to combat its antimicrobial activities [22,23]. However, compared to macrophages, neutrophils are more effective at invading and phagocytosing *S. aureus* biofilms *in vitro* and the transcriptional response of *S. aureus* biofilms during neutrophil invasion is relatively minimal [24]. Despite this, neutrophils remain a comparatively small proportion of recruited immune cells to sites of implant-associated infection, with the majority being myeloid derived suppressor cells [25]. In patients with prosthetic joint infection, the percentage of neutrophils is not different from those undergoing aseptic revision [26]. However, whether this is due to a lack of neutrophil recruitment or an infection-associated signal that arrests granulocyte precursors in an immature state remains unknown.

We previously observed that neutrophils associated with *S. aureus-*infected implants display a wandering migration phenotype [14]; we hypothesized that bacterial interference with neutrophil chemotactic signalling may be responsible. Given the key role of CXCR2 as a mediator of neutrophil recruitment and activation, we thus investigated the effects of either stimulating murine *Cxcr2* expression or antagonizing CXCR2 signalling during *S. aureus* implant-associated infection. These therapeutic approaches utilized an innate acute-phase inflammatory mediator mechanism of *Cxcr2* augmentation (Lipocalin-2; Lcn2) and a selective CXCR2 antagonist (AZD5069) currently undergoing clinical trials for treating several presentations of neutrophilic inflammation [27,28]. Lcn2, also called neutrophil gelatinase-associated lipocalin (NGAL), induces CXCR2 and has been shown in mice to be important for promoting neutrophil migration [17] and protective against bacterial infection [29,30]. Interestingly, Lcn2 also binds bacterial siderophores, thereby preventing iron uptake [31]; however, *S. aureus* Staphyloferrin A has evolved as an Lcn2-resistant siderophore [32]. On the other hand, pharmacological blockade of CXCR2 by AZD5069 has been shown to reduce neutrophil migration from systemic circulation into other compartments [28], yet is considered to have no effect on infection risk [33].

We hypothesized that Lcn2 treatment would lead to reduced bacterial burdens during our implant model of *S. aureus* infection while CXCR2 antagonism would inhibit neutrophil recruitment and therefore increase infection severity. Our results, however, demonstrate that both therapeutic approaches of modifying CXCR2 activity resulted in significantly increased *S. aureus* titres. A proposed explanation for these findings is the essential role macrophages play in both directly preventing bacterial dissemination as well as coordinating immune responses to *S. aureus* infection. Lcn2 was previously shown to skew macrophages to a deactivated phenotype during pneumococcal pneumonia infection in mice by inducing IL-10 production and skewing macrophage polarization in a STAT3-dependent manner [34]. Similar immune signalling responses have been described by us and others as responsible for *S. aureus* implant-associated infection establishment and persistence [4,25, 35]. While we did not detect significant differences in tissue IL-10 production (Fig. 1d) or *Il-10* expression (data not shown) from infected animals treated with Lcn2 compared to vehicle controls, it remains possible that even with the potentiating effects of Lcn2 on *Cxcr2* expression, its actions on macrophages and subsequent influence on immune responses that promote infection establishment and persistence may overshadow any protective effects on neutrophil chemotaxis and activation due to *Cxcr2* induction.

Antagonizing CXCR2 with AZD5069 concomitant with *S. aureus* biofilm infection of an indwelling catheter-associated surprisingly revealed a substantial upregulation of *Cxcr2* expression in the surrounding tissue. This has been observed using other CXCR2 antagonists in disparate disease models [36], and reveals a possible immune adaptive mechanism to increase neutrophil chemotaxis to the site of infection. Regardless, this potential adaptation was insufficient in offsetting CXCR2 antagonism, thus resulting in increased bacterial accumulation on the catheter and invasion into surrounding soft tissues. Furthermore, antagonization of CXCR2 led to the formation of thick fibrotic barriers at the biofilm-host tissue interface, similar to what has been observed previously in MyD88 knock-out mouse infections [37].

The regulation of CXCR2 expression and its role during disease is notably complicated and is often situation-dependent [38]. Similarly, the contribution of Lcn2 to disease states varies based on host and pathogen interactions [39]. These findings described here are clinically translatable as they emphasize the protective role CXCR2 plays in limiting *S. aureus* growth on medical implants. Future studies on the role of CXCR2-targeting virulence factors (i.e. ɣ-hemolysin AB, LukED, Staphopain A) during *S. aureus* biofilm-mediated infection may shed light on their contribution to thwarting neutrophil function via CXCR2 modulation. However, utilization of an inducer, such as Lcn2, to promote *Cxcr2* expression reveals that there is much more complexity in its protective role, and more experimentation will be necessary to identify an intervention for application into modern medical practice.

## Abbreviations

CXCR2: CXC chemokine receptor 2
H&E: hematoxylin and eosin
IL-10: interleukin 10
Lcn2: lipocalin-2
S. aureus: Staphylococcus aureus
